# A Protic Ionic Liquid Promoted Gel Polymer Electrolyte for Solid-State Electrochemical Energy Storage

**DOI:** 10.3390/ma17235948

**Published:** 2024-12-05

**Authors:** Jiaxing Liu, Zan Wang, Zhihao Yang, Meiling Liu, Hongtao Liu

**Affiliations:** Hunan Provincial Key Laboratory of Chemical Power Sources, College of Chemistry and Chemical Engineering, Central South University, Changsha 410083, China

**Keywords:** solid-state capacitor, protic ionic liquid, gel polymer electrolyte, electrochemical energy storage

## Abstract

This study presents the synthesis of a transparent, flexible gel polymer electrolyte (GPE) based on the protic ionic liquid BMImHSO_4_ and on polyvinyl alcohol (PVA) through solution casting and electrochemical evaluation in a 2.5 V symmetrical C/C electrical double-layer solid-state capacitor (EDLC). The freestanding GPE film exhibits high thermal stability (>300 °C), wide electrochemical windows (>2.7 V), and good ionic conductivity (2.43 × 10^−2^ S cm^−1^ at 20 °C). EDLC, using this novel GPE film, shows high specific capacitance (81 F g^−1^) as well as good retention above 90% of the initial capacitance after 4500 cycles. The engineered protic ionic liquid GPE is, hopefully, applicable to high-performance solid-state electrochemical energy storage.

## 1. Introduction

Safety is one of the most important factors when considering electrochemical energy storage applications. The liquid electrolytes commonly used in conventional electrochemical energy storage devices are prone to leaks and explosions when exposed to high pressure and temperature, leading to safety issues. Compared to liquid electrolytes, the gel polymer electrolytes (GPEs) are much safer in practice owing to their less volatile ingredients. GPEs generally contain three main components: polymer substrate frameworks (e.g., PVDF-HFP [1], PEO [2], PVA [3,4]), conducting salts (e.g., LiClO_4_ [5], LiPF_6_ [6]), and plasticizers based on esters [7,8]. The flexibility, ionic conductivity, and thermal and electrochemical stability of the GPEs can be optimized to meet application requirements by tuning the ratio of polymers to conducting salts to plasticizers [9].

Recently, ionic liquids (ILs) used as plasticizers in the fabrication of GPEs have attracted increasing attention in the context of catalysis [10], organic synthesis [11], and chemical analysis [12,13]. Compared to molecule-based ester plasticizers, ILs are composed entirely of organic cations and organic/inorganic anions, thus presenting definite ionic conductivity. Owing to their super electrochemical stability, thermal stability, flexibility, and low volatility compared to those of water and other common organic solvents, these novel environmentally friendly solvents have been widely used in Li-ion batteries [14,15,16], fuel cells [17], dye-sensitized solar cells [18], and supercapacitors [19,20,21]. ILs can be divided into three categories: aprotic ionic liquids, protic ionic liquids, and zwitterionic ionic liquids [22]. Among them, protic ionic liquids (PILs) are considered to be a plasticizer that can effectively enhance the performance of GPEs. In addition to the general characteristics of ILs, PILs provide free protons to enhance the ionic conductivity of the electrolyte [23,24]. Moreover, this kind of ILs can be prepared through simple synthetic procedures. Consequently, PILs have been attracting more attention from chemists. Since the first reported PIL in 1888 [25], PILs have been widely used in electrochemistry [26], chromatography [27], and self-assembly [28]. Shin et al. [29] prepared a freestanding and highly conductive GPE film with an IL/PEO weight fraction of 1.5 and demonstrated its excellent performance when used for lithium metal-polymer battery applications. Sanaz et al. [30] used the more conductive protic IL as a plasticizer and prepared PEO-based GPEs for a supercapacitor that exhibited both a high rate of capability and a stable cycle life. Saroj et al. [31] investigated the influence of IL plasticizer contents on the ionic conductivity of PVA-based GPEs and confirmed the dual roles of IL in improving both flexibility and conductivity of the GPEs.

In this paper, we combine BMImHSO_4_, a promising ionic liquid, with high-conductivity and electrochemically stable PILs and PVA to successfully prepare a freestanding GPE film to prepare GPEs. PVA is selected as the substrate framework because it is a polar polymer with better film-forming capabilities, water solubility, lower crystallinity than PEO, and higher ionic conductivity than PVDF-HFP [32]. The electrochemical and physical properties of the PIL/PVA gel polymer electrolyte are fully characterized and their potential advantages verified. The solid-state EDLC based on the targeted GPE are assembled and tested, demonstrating not only a high specific capacitance, but also a robust cyclic stability.

## 2. Materials and Methods

### 2.1. Chemicals

The following chemicals were used in this study: N–methylimidazole (99%, Alfa Aesar, Haverhill, MA, USA), bromobutane, acetonitrile, polyvinyl alcohol (PVA-124) (analytical pure, Sinopharm Chemical Reagent Co., Ltd., Shanghai, China), ethyl acetate (analytical pure, Tianjin Fuyu Fine Chemical Co., Ltd., Tianjin, China), sulfuric acid (analytical pure, Hengyang Kaixin Chemical Co., Ltd., Hengyang, China), diethyl ether, isopropyl alcohol (Tianjing Damao Chemical Co., Ltd., Tianjin, China), activated carbon (Nanjing Zhengsen Environmental Science and Technology Co., Ltd., Nanjing, China), acetylene black (Shenzhen Tianjiao Technology Development Co., Ltd., Shenzhen, China), 10% polytetrafluoro ethylene (10% PTFE, Shanghai Zhaozhi Nano Technology Co., Ltd., Shanghai, China). Stainless steel sieves (Hebei Yingda Wire Mesh Co., Ltd., Shijiazhuang, China) and CR2032 button battery shells (Shenzhen Tianjiao Technology Development Co., Ltd., Shenzhen, China) were also used. All chemicals were used as received.

### 2.2. Preparation of the Materials

#### 2.2.1. Synthesis of BMImHSO_4_

The ionic liquid was synthesized via the anionic substitution reaction between the intermediate BMImBr and the acid. Specifically, 7.9 mL N–methylimidazole was added to three flasks and stirred for 5 min at 35 °C. A total of 21 mL bromobutane was added to the solution in the flask dropwise over two hours, followed by a thermostatic reaction at 55 °C for 36 h. A white crystal was obtained after cooling the solution. The upper liquid was removed before washing the crystals with ethyl acetate (15 mL × 3) to get the BMImBr, which was subsequently dried at 50 °C for 12 h.

A total of 6.59 g of BMImBr was dissolved in 10 mL acetonitrile and stirred for 5 min at room temperature. Then, 1.6 mL of H_2_SO_4_ was added slowly. The temperature of the flask was kept below 20 °C using an ice–water bath. The reaction continued for 15 h at room temperature after the addition of H_2_SO_4_. The product was washed with anhydrous diethyl ether (15 mL × 4) to obtain a yellow, sticky liquid, which was finally vacuum-dried for 12 h.

#### 2.2.2. Preparation of PIL/PVA GPEs

GPEs were prepared through a conventional solution casting method [33,34]. PVA was added to the water and vigorously stirred for 30 min at 90 °C before adding BMImHSO_4_. The mixture was stirred for 15 min to obtain a homogeneous solution which was cooled down to room temperature in a vacuum oven to remove bubbles. Then, the solution was carefully casted onto clean glass molds, which were dried at room temperature to allow for the slow evaporation of most of the solvent before being transferred into a vacuum drying chamber for 12 h at 90 °C. The obtained off-plate GPE films, with a thickness of 0.5–1 mm, were translucent and freestanding.

#### 2.2.3. Preparation of Electrodes and Fabrication of EDLCs

Electric double-layer capacitors (EDLCs) with PIL-incorporated GPEs as electrolytes and activated carbon as electrode material were fabricated in this paper. Activated carbon (1600 m^2^ g^−1^, particle ~5 µm), acetylene black, and PTFE were used as received, the mass loading of the activated carbon was approximately 1.0 mg cm^−2^. Stainless steel sieves were used as current collectors. Activated carbon and acetylene black were mixed fully before adding PTFE. The mixture was dispersed in isopropyl alcohol to form a uniform slurry. The slurry was casted onto a stainless sieve and pressed into thin electrode flakes. After being vacuum-dried at 100 °C for 24 h, the flakes were cut into disks with a diameter of 14 mm. One GPE film was placed between two flakes with a similar mass of activated carbon and sealed into a CR2032 cell to obtain a symmetrical EDLC. The whole process was implemented in a glove box.

#### 2.2.4. Physiochemical Characterizations

The morphologies of the GPEs were observed using a scanning electron microscope (SEM, Nova NanoSEM 230, FEI Electron Optics B.V, Hillsboro, OR, USA). The samples were sprayed with gold before the test. The tensile test was performed using an electronic universal testing apparatus (INSTRON 5982, INSTRON, Norwood, MA, USA). The GPE samples for mechanical strength measurements were all cut into a membrane with a size of 5 cm × 1 cm × 0. 2 cm. The thermal stability of the GPEs was investigated using thermogravimetric/differential scanning calorimetry (TG/DSC SDTQ600, TA Instruments, Newcastle, DE, USA) at a heating rate of 10 °C min^−1^ under a nitrogen atmosphere from 20 °C to 650 °C. X-ray diffraction (XRD, JEOL-D/ruax2550PC, JEOL, Tokyo, Japan) was used to observe the crystalline structures of the GPEs, recording with Cu Kα radiation (40 kV, 30 mA, λ = 1.5418 Å) at a scan rate of 2° (2θ) min^−1^ from 5° to 70°.

#### 2.2.5. Electrochemical Measurements

The cyclic voltammetry (CV), electrochemical impedance spectroscopy (EIS), and galvanostatic charge–discharge (GCD) were performed on a CHI 660D electrochemical work station (CH Instruments, Bee Cave, TX, USA). Electrochemical stability and ionic conductivity tests used a symmetric cell comprising two stainless steel electrodes. The frequency range of the EIS test was 10 ^5^ Hz to 10 Hz for the GPEs and 10 ^5^ Hz to 0.01 Hz for the EDLC, and the used amplitude was 5 mV versus an open circuit potential. The voltage range of the GCD for the capacitor was 0 V to 2.5 V and the applied current density was 100 mA g^−1^.

## 3. Results and Discussion

**Figure 1:**
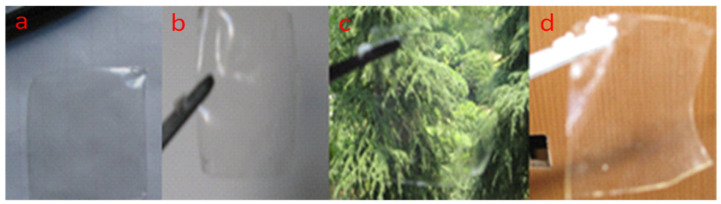
Optical photos of PIL/PVA GPE films with different PIL contents. (**a**) PIL 30 wt%; (**b**) PIL 50 wt%; (**c**) PIL 60 wt%; (**d**) PIL 70 wt%.

Figure 1 shows the optical photos of the GPEs with various contents of BMImHSO_4_, with BMImHSO_4_ fully characterized in our previous work [35]. The transparent films indicated the low crystallinity of GPEs, a property which is beneficial for ion transport. The transparent freestanding GPE films showed good mechanical strength even after a month of storage, demonstrating that GPEs have good stability.

As an intrinsic plasticizer, ionic liquid significantly decreases the insecurity caused by the volatility of conventional organic plasticizers [8,36,37]. On the other hand, excessive amounts of ionic liquid can weaken the forces between the polymer chains, leading to low mechanical strength of the substrate framework.

For further evaluation of the effect of PIL on the mechanical properties of GPEs, the tensile strength was tested, and the results are listed in Table 1. It can be seen that GPEs with a 70% content of PIL had the weakest mechanical properties. In addition, GPEs were saturated with liquid uptake when the PIL content reached 70%, a phenomenon which was proved by the trace PIL being observed on the surface of the GPE film. When the PIL content was further increased to 80%, GPE films could not remain self-supporting. Consequently, the PIL content should not exceed 70%.

**Figure 2:**
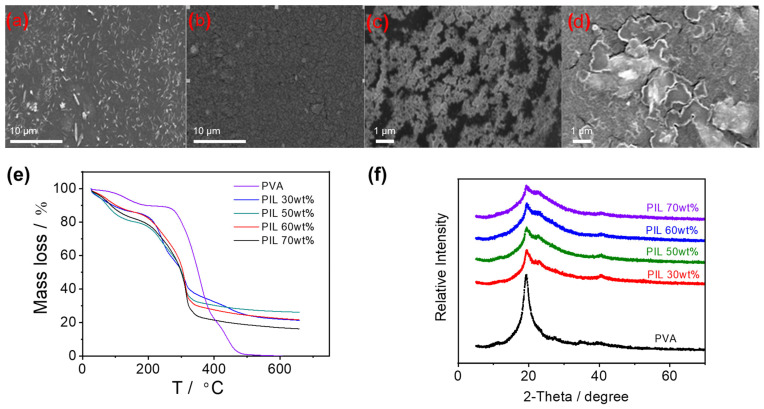
SEM of pure PVA and PIL/PVA GPEs. (**a**) upper surface of PVA film; (**b**) lower surface of PVA film; (**c**) upper surface of GPE (PIL, 60 wt%); (**d**) lower surface of GPE (PIL, 60 wt%); (**e**) TGA of PIL/PVA GPEs; (**f**) XRD of PIL/PVA GPEs.

Figure 2 compares the morphologies of pure PVA films and GPEs (PIL, 60 wt%). The PVA film was relatively more compact than the gel electrolyte (Figure 2a,b). The addition of IL considerably swelled the polymer, leading to a porous surface, which is theoretically beneficial for the movement of ions in the substrate. The different morphologies recorded in the upper surface (away from the glass mold) and the lower surface (close to the glass mold) of both films are the result of the difference in the solvent evaporation rate along the two surfaces. The solvent evaporated faster on the upper surface, forming larger pores. Conversely, the direct contact between the GPE film and the mold led to a drop in the evaporation rate of the solvent along the lower surface, resulting in a smoother morphology being observed.

The thermal stability of GPEs straightforwardly influences the performance of electrochemical devices [38,39]. In this case, TGA was used to investigate the stability of GPEs. As shown in Figure 2e, the mass loss below 120 °C was attributed to trance water in the PVA framework. Due to the hydrophilic nature of PIL, GPEs had a higher water content compared to pure PVA. From 120 °C upwards, the mass loss was caused by the dehydration and etherification of the hydroxyl groups in the PVA chains. According to Figure 2e, GPEs decomposed dramatically from 230 °C, followed by a greater mass loss around 300 °C as a result of PIL decomposition, whereas pure PVA remained stable at 270 °C due to stronger intramolecular and intermolecular hydrogen bonding. The results of TGA demonstrated that the incorporation of PIL was detrimental to the thermal stability of GPEs, thus the amount of PIL added should be controlled.

The ideal polymer solid-state electrolytes should have a considerable amorphous phase (low T_g_) convenient for ion transport, as well as low crystallinity which indicates a more amorphous structure. The XRD of the GPEs is shown in Figure 2f. The peak which appeared at 19.7° is the characteristic peak of crystalline PVA with numerous hydrogen groups. With respect to GPEs, the addition of PIL reduced the degree of crystallinity, leading to a reduction in peak intensity. This result matches well the TGA profiles. The decrease in PVA crystallization was supposed to increase the free volume of the GPEs, a phenomenon which facilitates ion migration. When the PIL content reached 60%, the peak intensity did not decrease further, indicating an inability to form more amorphous regions through the addition of PIL. To balance the conductive properties and mechanical strength of the film, the GPE with 60 wt% of PIL (459 N cm^−2^) was chosen to fabricate the EDLCs in this paper.

**Figure 3:**
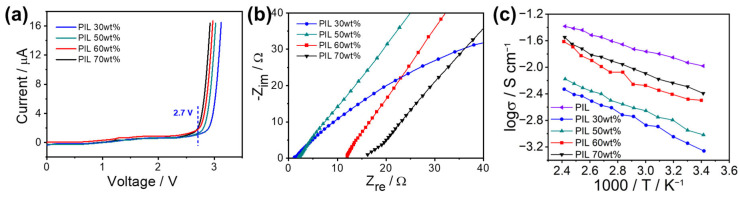
(**a**) Electrochemical stability of PIL/PVA GPEs; (**b**) EIS of PIL/PVA GPEs at room temperature from 10^5^ Hz to 10 Hz; (**c**) conductivity variations of pure PIL and PIL/PVA GPEs at different temperatures.

Electrochemical stability is another important property of electrolytes which determines the performance of electrochemical devices [40,41]. Nowadays, the electrochemical window of most IL-based electrolytes is above 3 V [1,42,43]. This high stability compared to that of other electrolytes is the decisive factor which has led to the promotion of its development in the field of electrochemistry. Figure 3a shows the linear sweep voltammetry of PIL/PVA GPEs with various PIL amounts. The GPEs remained stable up to 2.7 V, showing a relatively high electrochemical stability which is suitable for supercapacitors. However, similar to the results for TGA, excessive PIL amounts also impaired the electrochemical stability of the GPEs. This consequence confirms Tetsuya’s reports [44].

A series of experiments was carried out to investigate the ionic conductivity of the films. Equation (1) was used to calculate the conductivity of PIL and PIL/PVA GPEs at various temperatures.

(1) $$\sigma =\frac{d}{R_{b}S}$$

In this equation, *σ* is the conductivity of GPEs, *R_b_* is the bulk resistance of the GPEs, Ω, which can be obtained from Figure 3b, *d* is the thickness of the GPE films, cm, and *S* is the real testing area of the GPEs, cm^2^.

From the measurement results in Figure 3c, it can be seen that the ionic conductivity of the GPEs increased with temperature. This was accounted for by the larger free volume in the GPEs and by the faster ion migration at higher temperatures. Nevertheless, the GPE with 60 wt% of PIL was still able to reach a conductivity of 2.43 × 10^−2^ S cm^−1^ at 20 °C. The increase in GPE conductivity approached its maximum after the PIL content exceeded 60%, a finding which is consistent with the results from XRD. The activation energy of the GPEs with different contents of PIL remained essentially the same, i.e., 0.159 eV with 60 wt% of PIL, slightly lower compared to the 0.121 eV for pure PIL. The ionic conductivities of the GPEs based on different polymer matrices and/or PIL types were compared. As shown in Table 2, the high electrical conductivity of PIL and its good compatibility with PVA allowed the gel electrolyte to achieve high ionic conductivity at room temperature, even exceeding the performance of comparable electrolytes at elevated temperatures, also using commercial polymers as substrates.

**Figure 4:**
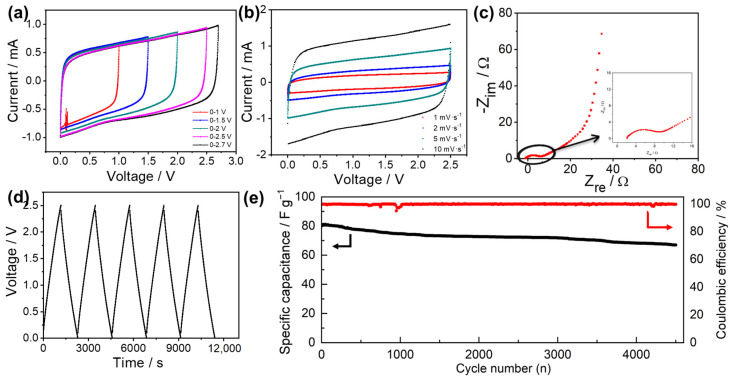
(**a**) CV of all-solid-state EDLCs under different potential ranges at 5 mV s^−1^ 0–1 V, 0–1.5 V, 0–2 V, 0–2.5 V, 0–2.7 V; (**b**) CV of all-solid-state EDLCs under different scan rates; (**c**) EIS of all-solid-state EDLC from 105 Hz to 0.01 Hz; (**d**) galvanostatic charge–discharge profiles of all-solid-state EDLCs at 100 mA g^−1^; (**e**) capacitance-retaining performance after 4500 charge–discharge cycles of the solid-state EDLC.

An EDLC with activated carbon as electrodes and a PIL/PVA GPE with 60 wt% of PIL as electrolytes were fabricated to test the electrochemical properties of the GPEs. Figure 4a,b shows the cyclic voltammetry of the EDLC at different voltages and scan rates, respectively. The rectangular curve obtained via a cyclic voltammetry test confirmed the typical characteristic of the electric double-layers formed between the electrodes and the gel electrolyte. The oxidation current increased slightly when the applied voltage increased to 2.7 V due to PIL decomposition (Figure 4a); therefore, the voltage range was set to 0–2.5 V. In Figure 4b, a high specific capacitance, 70.47 F g^−1^, was obtained at 10 mV s^−1^, which corresponds to 87% of that at 1 mV s^−1^. It indicates the excellent rate performance of the EDLC based on PIL-based GPEs.

Figure 4c shows the EIS profile of the EDLC. The whole resistance of the capacitor roughly contained two parts, 2.2 Ω of bulk resistance and 5.8 Ω of charge transfer resistance. The larger charge transfer resistance indicates that the main factor influencing the performance of the EDLC was the insufficient contact between the GPE and the electrode. This is a common problem involving electrochemical devices based on non-liquid electrolytes [50,51] and it can be resolved through casting electrolyte slurry directly on electrode flakes [52]. The vertically rising linear characteristic of the low frequency regions indicates the typical double-layer capacitance behavior of the activated carbon material.

Figure 4d shows the charge–discharge profile of the EDLC based on a PIL (PIL, 60 wt%) GPE at 100 mA g^−1^. The symmetrical triangles show a typical behavior with respect to EDLCs and good reversible ion adsorption/desorption along the surface of the electrodes. The IR drop was insignificant, representing the small internal resistance of the system. A discharge capacitance of 81 F g^−1^ was measured, which is similar to the results shown in Figure 4b. The EDLC presented good capacitance retention during the charge–discharge cycles and still retained 90% of the initial capacitance after 4500 cycles (Figure 4e). These competitive properties render this kind of PIL-based GPE a promising electrolyte for supercapacitor applications.

## 4. Conclusions

A promising PIL-incorporated polymer gel electrolyte was prepared successfully for solid-state EDLCs. By tuning the PIL content, the polymer gel electrolyte can form a flexible freestanding thin film. The PIL endowed the PIL/PVA electrolyte film with a high room-temperature ion conductivity of 2.43 × 10^−2^ S cm^−1^ in addition to promoting thermal stability (>230 °C) and the electrochemical window (>2.7 V), evidently suitable for high-performance supercapacitor applications. The assembled 2.5 V EDLC using the PIL/PVA gel polymer electrolyte exhibited both a high specific capacitance of 81 F g^−1^ and an excellent retention of 90% after 4500 cycles. Therefore, the PIL-based gel polymer electrolyte is promising for solid-state electrochemical energy storage.

## Figures and Tables

**Table 1:** Tensile strength (TS) of PIL/PVA GPEs.

PIL (wt%)	0	30	50	60	70
TS (N cm^−2^)	775	578	497	459	388

**Table 2:** Comparison of ionic conductivity between PIL/PVA and other PIL gel polymer electrolytes.

Polymers	PIL Types	Conductivity (S/cm)	Ref.
PVA	BMImHSO_4_	2.43 × 10^−2^ (20 °C)	This work
PDADMA	DEMATf	5.8 × 10^−3^ (100 °C)	[45]
PMMA	BMImTFSI	7.8 × 10^−4^ (28 °C)	[46]
PVDF-HFP	PyHSO_4_	4.29 × 10^−3^ (25 °C)	[47]
PVA	EHNH_2_H_2_PO_4_	6.2 × 10^−3^ (25 °C)	[48]
PVA/PMA	BMImTFSI	8.3 × 10^−4^ (60 °C)	[49]

## Data Availability

The original contributions presented in the study are included in the article, further inquiries can be directed to the corresponding author.

## References

[1] Ferrari S., Quartarone E., Mustarelli P., Magistris A., Fagnoni M., Protti S., Gerbaldi C., Spinella A. (2010). Lithium ion conducting PVdF-HFP composite gel electrolytes based on N-methoxyethyl-N-methylpyrrolidinium bis(trifluoromethanesulfonyl)-imide ionic liquid. J. Power Sources.

[2] Fang Z., Luo Y., Liu H., Hong Z., Wu H., Zhao F., Liu P., Li Q., Fan S., Duan W. (2021). Boosting the Oxidative Potential of Polyethylene Glycol-Based Polymer Electrolyte to 4.36 V by Spatially Restricting Hydroxyl Groups for High-Voltage Flexible Lithium-Ion Battery Applications. Adv. Sci..

[3] Saroj A.L., Singh R.K. (2011). Studies on ionic liquid 1-ethyl-3-methyl imidazolium ethylsulphate complexed with PVA. Phase Transit..

[4] He Q., Zhong Y., Li J., Chai S., Yang Y., Liang S., Chang Z., Fang G., Pan A. (2024). Constructing Kosmotropic Salt-Compatible PVA Hydrogels for Stable Zinc Anodes via Strong Hydrogen Bonds Preshielding Effect. Adv. Energy Mater..

[5] Xing F., Su F., Qin J., Wen P., Li Y., Zhang L., Ma J., Zheng S., Guo X., Wu Z.-S. (2023). 2D VOPO_4_ Pseudocapacitive Ultrafast-Charging Cathode with Multi-Electron Chemistry for High-Energy and High-Power Solid-State Lithium Metal Batteries. Adv. Energy Mater..

[6] Han L., Liao C., Liu Y., Yu H., Zhang S., Zhu Y., Li Z., Li X., Kan Y., Hu Y. (2022). Non-flammable sandwich-structured TPU gel polymer electrolyte without flame retardant addition for high performance lithium ion batteries. Energy Storage Mater..

[7] Zhang S., Liang T., Wang D., Xu Y., Cui Y., Li J., Wang X., Xia X., Gu C., Tu J. (2021). A Stretchable and Safe Polymer Electrolyte with a Protecting-Layer Strategy for Solid-State Lithium Metal Batteries. Adv. Sci..

[8] Ye X., Liang J., Hu J., Wu D., Li Y., Ouyang X., Zhang Q., Ren X., Liu J. (2023). An ultra-thin polymer electrolyte for 4.5 V high voltage LiCoO2 quasi-solid-state battery. Chem. Eng. J..

[9] Wang S., Jiang Y., Hu X. (2022). Ionogel-Based Membranes for Safe Lithium/Sodium Batteries. Adv. Mater..

[10] Sathyaseelan A., Elumalai V., Perumalsamy M., Ul Haq Liyakath Ali N., Sajeev A., Kim S.-J. (2024). Toward highly accessible Fe-N_4_ sites via rational design of metal chelated ionic liquids for ORR, OER and HER trifunctional electrocatalysis. Chem. Eng. J..

[11] Miao W., Chan T.H. (2006). Ionic-Liquid-Supported Synthesis:  A Novel Liquid-Phase Strategy for Organic Synthesis. Acc. Chem. Res..

[12] Li Y.L., Gross M.L., Hsu F.-F. (2005). Ionic-liquid matrices for improved analysis of phospholipids by MALDI-TOF mass spectrometry. J. Am. Soc. Mass Spectrom..

[13] Abraham M.H., Acree J.W.E. (2006). Comparative analysis of solvation and selectivity in room temperature ionic liquids using the Abraham linear free energy relationship. Green Chem..

[14] Huang G., Liao Y., Zhao X., Jin X., Zhu Z., Guan M., Li Y. (2023). Tuning a Solvation Structure of Lithium Ions Coordinated with Nitrate Anions through Ionic Liquid-Based Solvent for Highly Stable Lithium Metal Batteries. Adv. Funct. Mater..

[15] Liu Z., Hu Z., Jiang X., Wang X., Li Z., Chen Z., Zhang Y., Zhang S. (2022). Metal-Organic Framework Confined Solvent Ionic Liquid Enables Long Cycling Life Quasi-Solid-State Lithium Battery in Wide Temperature Range. Small.

[16] Li Y., Ding F., Shao Y., Wang H., Guo X., Liu C., Sui X., Sun G., Zhou J., Wang Z. (2024). Solvation Structure and Derived Interphase Tuning for High-Voltage Ni-Rich Lithium Metal Batteries with High Safety Using Gem-Difluorinated Ionic Liquid Based Dual-Salt Electrolytes. Angew. Chem. Int. Ed..

[17] Avid A., Ochoa J.L., Huang Y., Liu Y., Atanassov P., Zenyuk I.V. (2022). Revealing the role of ionic liquids in promoting fuel cell catalysts reactivity and durability. Nat. Commun..

[18] Can E., Uralcan B., Yildirim R. (2021). Enhancing Charge Transfer in Photocatalytic Hydrogen Production over Dye-Sensitized Pt/TiO_2_ by Ionic Liquid Coating. ACS Appl. Energy Mater..

[19] Liu H., He P., Li Z., Liu Y., Li J. (2006). A novel nickel-based mixed rare-earth oxide/activated carbon supercapacitor using room temperature ionic liquid electrolyte. Electrochim. Acta.

[20] Selvam S., Park Y.-K., Yim J.-H. (2022). Design and Testing of Autonomous Chargeable and Wearable Sweat/Ionic Liquid-Based Supercapacitors. Adv. Sci..

[21] Liang Z., Zhao C., Zhao W., Zhang Y., Srimuk P., Presser V., Feng G. (2021). Molecular Understanding of Charge Storage in MoS_2_ Supercapacitors with Ionic Liquids. Energy Environ. Mater..

[22] Lu X., Burrell G., Separovic F., Zhao C. (2012). Electrochemistry of Room Temperature Protic Ionic Liquids: A Critical Assessment for Use as Electrolytes in Electrochemical Applications. J. Phys. Chem. B.

[23] Duan Z., Gu Y., Zhang J., Zhu L., Deng Y. (2006). Protic pyridinium ionic liquids: Synthesis, acidity determination and their performances for acid catalysis. J. Mol. Catal. A Chem..

[24] Pundir S.S., Mishra K., Rai D.K. (2015). Poly(vinyl)alcohol/1-butyl-3-methylimidazolium hydrogen sulfate solid polymer electrolyte: Structural and electrical studies. Solid State Ion..

[25] Gabriel S. (1888). Ueber vinylamin und bromäthylamin(II.). Berichte Dtsch. Chem. Ges..

[26] Sun J., Liu Z., Zhou H., Cao M., Cai W., Xu C., Xu J., Huang Z. (2024). Ionic Liquids Modulating Local Microenvironment of Ni–Fe Binary Single Atom Catalyst for Efficient Electrochemical CO_2_ Reduction. Small.

[27] Poole C.F. (2004). Chromatographic and spectroscopic methods for the determination of solvent properties of room temperature ionic liquids. J. Chromatogr. A.

[28] Bryant S.J., Atkin R., Gradzielski M., Warr G.G. (2020). Catanionic Surfactant Self-Assembly in Protic Ionic Liquids. J. Phys. Chem. Lett..

[29] Shin J.-H., Henderson W.A., Passerini S. (2005). PEO-Based Polymer Electrolytes with Ionic Liquids and Their Use in Lithium Metal-Polymer Electrolyte Batteries. J. Electrochem. Soc..

[30] Ketabi S., Le Z., Lian K. (2011). EMIHSO4-Based Polymer Ionic Liquid Electrolyte for Electrochemical Capacitors. Electrochem. Solid-State Lett..

[31] Saroj A.L., Singh R.K. (2012). Thermal, dielectric and conductivity studies on PVA/Ionic liquid [EMIM][EtSO_4_] based polymer electrolytes. J. Phys. Chem. Solids.

[32] Ayesh A.I., Mohsin M.A., Haik M.Y., Haik Y. (2012). Investigations on electrical properties of poly(vinyl alcohol) doped with 1-methyl-3-n-decyl-imidazolium bromide ionic liquid. Curr. Appl. Phys..

[33] Ye T., Zou Y., Xu W., Zhan T., Sun J., Xia Y., Zhang X., Yang D. (2020). Poorly-crystallized poly(vinyl alcohol)/carrageenan matrix: Highly ionic conductive and flame-retardant gel polymer electrolytes for safe and flexible solid-state supercapacitors. J. Power Sources.

[34] Cheng H., Zhu C., Huang B., Lu M., Yang Y. (2007). Synthesis and electrochemical characterization of PEO-based polymer electrolytes with room temperature ionic liquids. Electrochim. Acta.

[35] Wang Z., Liu Y., Liu H. (2012). A novel protic ionic liquid/silica gel electrolyte and its properties. Sci. Sin. Chim..

[36] Jaipal Reddy M., Sreekanth T., Subba Rao U.V. (1999). Study of the plasticizer effect on a (PEO + NaYF4) polymer electrolyte and its use in an electrochemical cell. Solid State Ion..

[37] Rajendran S., Bama V.S. (2010). A study on the effect of various plasticizers in poly(vinyl acetate)-poly(methyl methacrylate) based gel electrolytes. J. Non-Cryst. Solids.

[38] Kawamura T., Kimura A., Egashira M., Okada S., Yamaki J.-I. (2002). Thermal stability of alkyl carbonate mixed-solvent electrolytes for lithium ion cells. J. Power Sources.

[39] Ravdel B., Abraham K.M., Gitzendanner R., DiCarlo J., Lucht B., Campion C. (2003). Thermal stability of lithium-ion battery electrolytes. J. Power Sources.

[40] Jiang X., Liu Z., Liu W., Yu D., Zhang J., Wang X., Zhang Y., Zhang S. (2024). Physical ionogels with only 2 wt% gelators as efficient quasi-solid-state electrolytes for lithium batteries. Matter.

[41] Zhang S., Chen W., Hao W., Li D., Zhang C., Yu F., Chen Y. (2024). A composite gel polymer electrolyte by incorporating modified POSS endowing inorganic-rich SEI formation and stable cycle life for lithium metal batteries. Chem. Eng. J..

[42] Weng C., Ma L., Wang B., Meng F., Yang J., Ji Y., Liu B., Mai W., Huang S., Pan L. (2024). Single-solvent ionic liquid strategy achieving wide-temperature and ultra-high cut-off voltage for lithium metal batteries. Energy Storage Mater..

[43] Zhou T., Zhao Y., Choi J.W., Coskun A. (2021). Ionic Liquid Functionalized Gel Polymer Electrolytes for Stable Lithium Metal Batteries. Angew. Chem. Int. Ed..

[44] Tsuda T., Nohira T., Nakamori Y., Matsumoto K., Hagiwara R., Ito Y. (2002). A highly conductive composite electrolyte consisting of polymer and room temperature molten fluorohydrogenates. Solid State Ion..

[45] Rao J., Wang X., Yunis R., Ranganathan V., Howlett P.C., MacFarlane D.R., Forsyth M., Zhu H. (2020). A novel proton conducting ionogel electrolyte based on poly(ionic liquids) and protic ionic liquid. Electrochim. Acta.

[46] Tamilarasan P., Ramaprabhu S. (2014). Stretchable supercapacitors based on highly stretchable ionic liquid incorporated polymer electrolyte. Mater. Chem. Phys..

[47] Vázquez-Fernández I., Bouzina A., Raghibi M., Timperman L., Bigarré J., Anouti M. (2020). Influence of hydrophilic/hydrophobic protic ionic liquids (PILs) on the poly(vinylidene fluoride) (PVDF-ionic liquid) membrane properties. J. Mater. Sci..

[48] Vázquez-Fernández I., Raghibi M., Bouzina A., Timperman L., Bigarré J., Anouti M. (2021). Protic ionic liquids/poly(vinylidene fluoride) composite membranes for fuel cell application. J. Energy Chem..

[49] Thanganathan U., Nogami M. (2015). Investigations on effects of the incorporation of various ionic liquids on PVA based hybrid membranes for proton exchange membrane fuel cells. Int. J. Hydrogen Energy.

[50] Tien C., Liang W., Kuo P., Teng H. (2008). Electric double layer capacitors with gelled polymer electrolytes based on poly(ethylene oxide) cured with poly(propylene oxide) diamines. Electrochim. Acta.

[51] Liu H., Liu Y., Li J. (2010). Ionic liquids in surface electrochemistry. Phys. Chem. Chem. Phys..

[52] Pandey G.P., Hashmi S.A., Kumar Y. (2010). Performance Studies of Activated Charcoal Based Electrical Double Layer Capacitors with Ionic Liquid Gel Polymer Electrolytes. Energy Fuels.

